# Zizhu Ointment Inhibits IgE–FcεRI Signaling‐Mediated Mast Cell Degranulation to Promote the Healing of Diabetic Foot Ulcers

**DOI:** 10.1155/jdr/9225968

**Published:** 2026-04-27

**Authors:** Jian Sun, Xiaoqian Zhang, Meijie Yuan, Xiao Yang, Lei Wang, Hongshuo Shi, Guobin Liu, Yang You

**Affiliations:** ^1^ Department of Vascular Surgery, Shuguang Hospital Affiliated to Shanghai University of Traditional Chinese Medicine, Shanghai, China, shutcm.edu.cn; ^2^ The Second Affiliated Hospital of Shandong University of Traditional Chinese Medicine, Jinan, China, bucm.edu.cn; ^3^ Shandong University of Traditional Chinese Medicine, Jinan, China, sdutcm.edu.cn

**Keywords:** degranulation, diabetic foot ulcer, igE-fcεRI signaling, mast cell, zizhu ointment

## Abstract

Diabetic ulcers, a serious complication associated with diabetes, present a significant therapeutic challenge due to their recurrent and persistent chronic inflammation. A crucial factor contributing to this sustained inflammation is mast cell degranulation. This study examined the effects of the traditional Chinese herbal formula Zizhu ointment (ZZO) on mast cell degranulation in diabetic ulcers, as well as its underlying mechanisms in promoting wound healing. Analysis of clinical specimens revealed that ZZO significantly inhibited mast cell degranulation in diabetic ulcer wounds. In a diabetic mouse wound model, ZZO was observed to suppress mast cell degranulation, decrease the expression of TNF‐α and MMP‐9, and facilitate wound healing. Cellular experiments demonstrated that ZZO inhibited IgE/DNP cross‐linking‐mediated degranulation, calcium (Ca^2+^) influx, and the release of β‐hexosaminidase, histamine (HIS), and TNF‐α in both human HMC‐1 and murine P815 mast cells. Furthermore, ZZO was found to block the IgE/DNP cross‐linking‐mediated activation of FcεRI‐proximal signaling pathways (LYN/SYK/PLCγ) and inflammatory cascades (IKK/NFκB and MAPKs). We hypothesize that ZZO facilitates the healing of diabetic ulcers by stabilizing mast cells and inhibiting their degranulation, thereby reducing the release of inflammatory mediators and promoting wound repair.

## 1. Introduction

Diabetic foot ulcer (DFU), is one of the most common and severe chronic complications among diabetic patients, representing a significant global health challenge [1]. According to projections by the International Diabetes Federation (IDF), the global diabetic population is anticipated to reach 783 million by 2045. Alarmingly, the lifetime incidence rate of DFU in this population is as high as 34%, indicating that, on average, 34 out of every 100 diabetic patients may develop foot ulcers during their lifetime [2]. This high prevalence not only causes substantial physical suffering and financial burdens for patients but also places a considerable strain on healthcare resources and public health systems worldwide [3, 4]. Consequently, DFU has become an urgent public health priority, necessitating immediate attention and coordinated interventions from healthcare providers, policymakers, and society at large to mitigate its extensive impact [5, 6].

Recent research has elucidated the complex role of mast cell degranulation in the pathogenesis and progression of DFU [7, 8]. Mast cells, which are immune cells extensively distributed in the skin, mucous membranes, and perivascular regions, are characterized by their cytoplasmic granules that are rich in bioactive mediators such as histamine (HIS), proteases (including tryptase and chymase), and heparin [9]. Under normal physiological conditions, these cells contribute to immune defense, the regulation of inflammation, and tissue repair [10, 11]. However, within the pathological microenvironment of DFUs, mast cell degranulation may become dysregulated [12]. During the initial stages of ulcer development, localized tissue damage and inflammatory responses induced by hyperglycemia lead to excessive mast cell degranulation [13]. The release of HIS results in local vasodilation and increased vascular permeability, which exacerbates tissue edema and hypoxia‐key obstacles to effective healing. Simultaneously, proteases degrade components of the extracellular matrix (ECM), thereby compromising tissue integrity and hindering reparative processes [14]. Additionally, degranulation enhances localized immune activation by recruiting further inflammatory cells, such as neutrophils and macrophages. This pathological cascade not only maintains a pro‐inflammatory environment but also disrupts the spatiotemporal coordination of wound healing, making DFUs resistant to standard therapies. Targeting mast cell‐mediated pathways, such as protease inhibition and HIS receptor blockade, may therefore offer a promising therapeutic strategy to interrupt the cycle of chronic inflammation and restore normal healing processes in the management of DFUs [15].

In China, traditional Chinese medicine (TCM) topical agents for external surgical use have shown significant efficacy in managing chronic wounds, particularly DFUs, by enhancing local microcirculation, resolving inflammation, and accelerating tissue repair, thus reducing healing time and complications [16, 17]. Grounded in TCM surgical theory and enriched by clinical expertise, our team developed zizhu ointment (ZZO), a multicomponent formulation, that synergistically modulates the immune microenvironment of DFUs [18]. Preclinical studies have elucidated that its mechanism of action involves the suppression of pro‐inflammatory mediators, such as TNF‐α and NLRP3, stabilization of ECM, and facilitation of inflammatory resolution. These actions effectively address pathological features, including chronic inflammation and impaired repair process [19, 20]. By integrating principles of TCM with contemporary molecular insights, ZZO exemplifies the potential of traditional formulations to contribute to evidence‐based wound care. Its offers a standardized therapeutic strategy aimed at improving clinical outcomes in the management of diabetic wounds (DWs), thereby highlighting the enduring relevance of TCM in modern medical practice.

The primary objective of this study is to investigate the specific effects of ZZO on mast cell degranulation in DWs and to elucidate its underlying therapeutic mechanisms in DFU treatment by analyzing expression changes of related signaling pathways. By systematically exploring how this TCM formulation modulates pathological mast cell activity and downstream molecular cascades, we aim to establish a robust theoretical foundation for its clinical application, ultimately advancing the integration of TCM‐based topical therapies into modern evidence‐based medical practices and expanding their therapeutic potential in chronic wound management.

## 2. Materials and Methods

### 2.1. Antibodies and Reagents

Anti‐SYK (#13198), anti‐p‐SYK (#2710), anti‐LYN (#2796), anti‐p‐LYN (#2731), anti‐PLCγ1 (#5690), anti‐p‐PLCγ1 (#2821), anti‐PI3K (#4292), anti‐p‐PI3K (#17366), anti‐AKT (#9272), anti‐p‐AKT (#4060), anti‐IKKα (#61294), anti‐p‐IKKα/β (#2078), anti‐NFκB (#8242), anti‐p‐NFκB (#3033), anti‐JNK (#9252), anti‐p‐JNK (#4668), anti‐ERK (#4695), anti‐p‐ERK (#4370), anti‐P38 (#4695), anti‐p‐P38 (#9212), and goat anti‐rabbit IgG horseradish‐peroxidase (HRP) were purchased from Cell Signaling Technology. Anti‐GAPDH (10494‐1‐AP), anti‐MMP9 (10375‐2‐AP), anti‐GAPDH (17590‐1‐AP), and HRP‐conjugated anti‐mouse (SA00001‐1) were obtained from ProteinTech Group, Inc.

Anti–2,4‐dinitrophenyl (DNP)–IgE (D8406), DNP–HSA (A6661), Tyrode’s buffer (T2397), and the β‐hexosaminidase substrate 4‐nitrophenyl N‐acetyl‐β‐D‐glucosaminide (N9376) were purchased from Sigma–Aldrich.

### 2.2. Preparation of the ZZO and Its Extracts

ZZO is a pharmaceutical preparation (Approval Number 130301) developed by Shuguang Hospital Affiliated to Shanghai University of TCM. The herbal constituents were sourced from Shanghai Kangqiao Chinese Medicine Tablet Co., Ltd., and conformed to established quality control standards. The ointment was produced by Wuhan Mayinglong Pharmaceutical Group Co., Ltd. It comprises six active ingredients: *Astragalus membranaceus* (1.5%), cinnabaris (3.5%), donkey‐hide gelatin (2.5%), *Arnebia/Lithospermum* root (1.5%), dragon’s blood resin (3%), and borneol (0.5%), in addition to excipients such as white petrolatum and lanolin.

For experimental applications involving cells, the alcohol extract of the ZZO was prepared as follows: 150 g *Arnebia/Lithospermum* root, 350 g cinnabaris, 300 g dragon’s blood resin, 150 g *Astragalus membranaceus*, 250 g donkey‐hide gelatin, and 50 g borneol were mixed with 10 volumes of 80% ethanol (v/w), soaked for 24 h, heated to boiling over high heat, and then simmered at low heat for 1 h before vacuum filtration. The residue was re‐extracted with 10 volumes of 80% ethanol overnight, followed by repeating the heating and filtration steps. The combined filtrates were concentrated via rotary evaporation until ethanol‐free, yielding the final alcohol extract, which was stored at 4°C and sterilized through a 0.22 μm micropore filter prior to application.

### 2.3. Clinical Specimens

The study protocol was approved by the Ethics Committee of Shanghai University of TCM (Approval No. 2024‐1443‐026‐01) and complied with the Declaration of Helsinki. Participants included DFU patients and healthy controls. Healthy controls (*n* = 6) were individuals without diabetes or dermatological disorders, and nonrepairable wound‐edge skin tissues were obtained during post‐traumatic debridement.

A total of 46 patients with non‐ischemic DFU were enrolled. All participants first received standard wound care, including routine debridement, infection control, and anti‐inflammatory management. Thereafter, ZZO was applied topically according to the wound‐healing stage following a standardized, widely used protocol in TCM clinical practice. In the inflammatory phase, a thick layer of ZZO (~4 mm) was evenly applied to the ulcer surface, firmly pressed, and covered with sterile gauze once daily. When exudation decreased and healthy granulation tissue appeared, indicating entry into the granulation phase, a medium‐thickness layer (~2 mm) was applied with moderate pressure, and dressings were changed every 2 days. In the epithelialization phase, a thin layer of ZZO (~1 mm) was applied with light pressure, and dressings were changed every 3 days until healing was complete. Ulcer tissue specimens were collected at baseline and after 8 weeks of treatment during debridement performed under local anesthesia. Written informed consent was obtained from all participants.

### 2.4. Mice

A cohort of 36 male C57/BL6J mice, aged 8 weeks and weighing between 23 and 28 g, with a specific pathogen‐free (SPF) status, was procured from Shanghai Jihui Laboratory Animal Breeding Co., Ltd. (Shanghai, China). The mice were housed in an SPF‐grade facility at the Experimental Animal Center of Shanghai University of TCM, where environmental conditions were meticulously controlled: temperature maintained at 23 ± 2°C, relative humidity at 55% ± 5%, and a 12 h light/dark cycle. Throughout the study, mice had ad libitum access to standard chow and water, with bedding replaced every 3 days to ensure hygienic conditions [18]. Prior to the commencement of formal experiments, all mice underwent a 1‐week acclimatization period to minimize stress and stabilize physiological parameters. All experimental procedures were conducted in strict accordance with national guidelines and the institutional animal care regulations of Shanghai University of TCM. The study protocol was approved by the Animal Ethics Committee of Shanghai University of TCM (Approval No. PZSHUTCM2212140011).

### 2.5. The Wound Model and Treatment

The DW model in mice was developed based on a previously established mouse diabetic model [18]. Initially, the mice were fed a high‐fat diet for 8 weeks. Subsequently, they received intraperitoneal injections of a 1.5% streptozotocin (STZ) solution, prepared with citric acid and sodium citrate at a dosage of 40 mg/kg, administered to the abdominal region for five consecutive days. One week post‐STZ injection, random blood glucose levels were assessed. The model was deemed stable when blood glucose levels reached 16.7 mmol/L and remained consistent for an additional week. Following this, the dorsal hair of the mice was shaved, and anesthesia was induced using isoflurane gas. A circular area with a diameter of 1.5 cm was delineated on the dorsal skin, and the epidermal layer was completely excised. Mice in the control group were maintained on a standard diet and did not receive STZ treatment. Concurrently, a standard wound model was established in the mice using the aforementioned procedures.

In this animal study, a total of 36 mice were randomly divided into three experimental groups (*n* = 12/group). The control and DW groups were both treated with normal saline. The DW + ZZO group received topical treatment with ZZO ointment at a dose of 0.032 g/cm^2^ applied uniformly to the wound surface once daily. This dosage was achieved by preparing a medicated gauze, where 3.2 g of ZZO (equivalent to ~3 mL in volume) was evenly spread over a 10 cm × 10 cm gauze, ensuring consistent drug concentration and application.

### 2.6. Wound Healing Assay

When determining the WHR, standardized photography of the mouse wounds was first performed on days 0, 3, 7, and 14 post‐intervention. Subsequently, the captured wound images were analyzed using ImageJ software to quantify the wound area (WA) at each time point. The healing rate was calculated using the formula: WHR_*x*_ = ([WA_0_ − WA_*x*_]/WA_0_) × 100%, where WA_0_ represents the initial WA, WA_*x*_ denotes the WA at specific time points (days 3, 7, or 14), and WHR_*x*_ indicates the healing rate at the corresponding time point.

### 2.7. Hematoxylin and Eosin (H&E) Staining

Collected tissue samples were fixed in 4% paraformaldehyde (Beyotime, China) for 48 h. Fixed specimens then underwent sequential histological processing, including embedding, dehydration, clearing, paraffin infiltration, embedding, sectioning (4 μm thickness), baking, dewaxing, rehydration, H&E staining (Beyotime, China), clearing, and mounting. Histological images were captured using a light microscope and archived for quantitative analysis using the ImageView image analysis system.

### 2.8. Toluidine Blue (TB) Staining

TB staining reagents for tissue samples were procured from Shanghai Gefan Biotechnology Co., Ltd. (Shanghai, China). Paraffin‐embedded tissue sections underwent deparaffinization and rehydration through a graded ethanol series. Subsequently, the sections were then incubated in a TB staining solution for 30 min at room temperature, followed by three rinses with distilled water, each lasting 2 min. Differentiation was achieved using 95% ethanol until mast cell granules displayed a distinct purplish‐blue coloration against a pale blue background. The sections were then dehydrated in absolute ethanol, cleared in xylene, and mounted with neutral resin.

TB staining protocol for cells was performed as follows: cell suspensions were smeared onto glass slides and air‐dried. Slides were incubated with cell‐specific TB staining solution (Solarbio, China) for 5 min. An equal volume of distilled water was then added dropwise to the smears and mixed gently, followed by additional staining for 15 min. The slides were washed twice with distilled water (30 s per wash), air‐dried, and mounted with neutral resin under a coverslip. Stained cells were examined under a light microscope for morphological analysis.

### 2.9. Immunohistochemical (IHC) Staining

The paraffin‐embedded tissue samples were prepared through a process of continuous sectioning, followed by overnight heating at 60°C. The samples were then washed with a toluene‐based alcohol solution, subjected to gradient ethanol lysis, and underwent antigen retrieval. This was followed by incubation with primary and secondary antibodies, DAB staining, counterstaining with hematoxylin, and successive ethanol dehydration, culminating in embedding with neutral resin. Subsequent to these preparations, observation and imaging were conducted using optical microscopy. The optical density (OD) of the images was analyzed using ImageJ software to assess differences in antigen expression among various samples.

### 2.10. Bioinformatic Analysis of Immune Cell Composition

The gene expression dataset GSE134431 was sourced from the National Center for Biotechnology Information Gene Expression Omnibus (GEO) database (https://www.ncbi.nlm.nih.gov/geo/), comprising transcriptomic profiles of eight normal skin specimens from diabetic foot skin (DFS) and 13 ulcer specimens from DFU patients. The raw data were normalized using the robust multiarray average (RMA) algorithm to generate expression matrices for subsequent analysis. The composition of immune cell composition was quantified using the R implementation of CIBERSORT, employing the LM22 transcriptional signature matrix, which includes 22 immune cell subtypes, such as macrophage subsets, T cells, NK cells, mast cells, B cells, dendritic cells, monocytes, plasma cells, neutrophils, and eosinophils. To ensure robustness, 1000 Monte Carlo permutations were conducted.. Samples with a CIBERSORT *p*‐value <0.05 were retained for subsequent analyses. Differential abundance of 22 immune cell subtypes between DFS and DFU groups was assessed using the Wilcoxon rank‐sum test (*p* < 0.05 considered significant). Statistical analyses were performed in R (version 4.3.1), and results were visualized using the packages “corrplot,” “vioplot,” “ggplot2,” and “glmnet” to generate correlation heatmaps, violin plots, and regression models [21].

Transcriptome sequencing of wound tissues from model mice was performed by Shanghai OE Biotech Co., Ltd. For day 3 post‐treatment specimens, samples included the control group (D3C1), the DW group (D3DW1, D3DW2, and D3DW3), and the DW + ZZO group (D7DWZ1, D7DWZ2, and D7DWZ3). Day 7 post‐treatment specimens comprised the control group (D7C1, D7C2, and D7C3), the DW group (D7DW1 and D7DW2), and the DW + ZZO group (D7DWZ1, D7DWZ2, and D7DWZ3). Sequencing data were analyzed using the CIBERSORT algorithm in R to quantify immune cell composition. The reference marker gene expression profile matrix was based on a widely used mouse immune cell signature matrix [22].

### 2.11. Cell Lines and Cell Culture

In this study, the human mast cell line HMC‐1 was purchased from Yaji Biotechnology Co., Ltd. (Yaji Cell Center, Shanghai, China), the mouse myeloid mastocytoma cell line P815 was purchased from the Institute of Cell Biology (Shanghai, China). HMC‐1 and P815 were cultured in IMDM (Gibco, USA) and DMEM (Gibco), respectively, which supplemented with 10% (v/v) fetal bovine serum (FBS) (Gibco) and 1% penicillin/streptomycin (Solarbio, China), maintained at 37°C in a 5% CO_2_ atmosphere.

### 2.12. CCK8 Assay

After HMC‐1 and P815 cells reached the logarithmic growth phase, they were seeded into 96‐well plates at a density of 5 × 10^3^ cells per well using medium supplemented with 10% FBS. Following 24 h of incubation, the culture medium was replaced with fresh medium containing ZZO at concentrations of 0, 50, 100, 200, 400, 800, 1600, and 3200 μg/mL, with six replicate wells per concentration. After 48 h of drug treatment, 10% CCK‐8 solution (Beyotime, China) was added to each well and thoroughly mixed, followed by incubation at 37°C for 4 h. The OD was measured at dual wavelengths of 490 and 630 nm using a microplate reader. The cell proliferation inhibition rate (%) was calculated according to the formula: Inhibition rate (%) = (1 − [OD experimental group − OD blank group]/[OD control group − OD blank group]) × 100%.

### 2.13. Activation of Mast Cell Degranulation

After HMC‐1 and P815 cells reached the logarithmic growth phase, mast cell activation was induced using an IgE/DNP crosslinking protocol. Briefly, cells were first sensitized with 200 ng/mL anti‐DNP IgE overnight, followed by three washes with PBS to remove unbound IgE. To assess the inhibitory effect of ZZO, an additional experimental group was included in which cells were pretreated with 200 μg/mL ZZO for 2 h after IgE sensitization and immediately before DNP–HSA stimulation. Subsequently, IgE‐sensitized cells (with or without ZZO pretreatment) were stimulated with 100 ng/mL DNP–HSA at 37°C for 4 h to induce FcεRI‐mediated mast cell activation and degranulation. Thus, three parallel conditions were established: (i) untreated control, (ii) IgE/DNP‐activated group, and (iii) ZZO‐pretreated + IgE/DNP group. All downstream assays—including TB‐based degranulation assessment, cytosolic Ca^2+^ imaging, β‐hexosaminidase release, HIS quantification, and TNF‐α ELISA—were performed under these conditions to evaluate the inhibitory effects of ZZO on IgE‐triggered mast cell activation.

### 2.14. Cytosolic Calcium (Ca^2+^) Staining

Cytosolic Ca^2+^ concentrations were quantified utilizing the Fluo‐4 Ca^2+^ Assay Kit (Beyotime, China). Following cell collection, the cells underwent two washes with phosphate‐buffered saline (PBS) and were subsequently incubated with 5 μM Fluo‐4 in a dark environment at 37°C for 30 min. Post‐incubation, the cells were washed once with PBS and resuspended in assay buffer. The prepared samples were then divided into two aliquots for subsequent fluorescence microscopy and flow cytometric analysis. Fluorescence imaging was conducted using an IX71 fluorescence microscope (Olympus Corporation, Tokyo, Japan). Prior to imaging, the cells were transferred to a 96‐well plate, and bright‐field images were merged with green fluorescence images (488 nm excitation) captured under the microscope. Flow cytometric analysis was performed using a CytoFLEX Flow Cytometer (Beckman Coulter, Brea, CA, USA), wherein the mean fluorescence intensity (MFI) of green fluorescence (488 nm excitation) was quantified for each sample.

### 2.15. Detection of β‐hexosaminidase Release

After the completion of the mast cell degranulation activation experiment, the samples were centrifuged to separate cells and supernatant. The cell pellets were lysed in Tyrode’s buffer containing 1% Triton X‐100 for 30 min, followed by centrifugation to collect the lysate supernatant. The cell culture supernatant and lysate supernatant were then mixed with an equal volume of substrate solution (1 mM 4‐nitrophenyl N‐acetyl‐β‐D‐glucosaminide in 0.1 M citrate buffer, pH 4.5) and incubated at 37°C for 1 h. The reaction was terminated by adding 0.1 M Na_2_CO_3_/NaHCO_3_ buffer (pH 10), and the absorbance was measured at 405 nm using a microplate reader (EXL‐800, BioTek). The β‐hexosaminidase release rate (%) is calculated using the following formula: % release = (supernatant OD/[supernatant OD + lysate OD]) × 100%.

### 2.16. Measurement of HIS and TNF‐α

The HIS content in the cell culture supernatants of activated HMC‐1 and P815 cells were quantified using a commercially available HIS ELISA Kit (Cat. No. E‐EL‐0032, Elabscience Biotechnology Co., Ltd., Wuhan, China), following the manufacturer’s protocol. The TNF‐α content in the cell culture supernatants of activated HMC‐1 and P815 cells were quantified using EasyGo! Human TNF‐α One‐Step ELISA Kit (Cat. No. EK182EGB, Multisciences, Hangzhou, China) and EasyGo! Mouse TNF‐α One‐Step ELISA Kit (Cat. No. EK282EGB, Multisciences, Hangzhou, China), respectively, following the manufacturer’s protocol.

### 2.17. Western Blot Analysis

Following activation, HMC‐1 and P815 cells were lysed using cell lysis buffer (Beyotime, China) supplemented with protease inhibitor (Beyotime, China) and phosphatase inhibitor (Beyotime, China) on ice for 30 min. Lysates were centrifuged at 12,000 × *g* for 15 min at 4°C, and supernatants were collected for protein quantification using the BCA Protein Assay Kit (Beyotime, China). Equal amounts of protein (20–30 μg per lane) were separated by 10% SDS–PAGE and transferred to PVDF membranes (Millipore, Billerica, MA, USA) using a wet transfer system at 200 mA for 2 h. Membranes were blocked with 5% non‐fat milk in TBST (tris‐buffered saline containing 0.1% tween‐20) for 1 h at room temperature and subsequently incubated overnight at 4°C with primary antibodies diluted in blocking buffer. After three TBST washes, membranes were probed with HRP‐conjugated secondary antibodies (Cell Signaling Technology, Danvers, MA, USA) for 1 h at room temperature. Protein bands were visualized using enhanced chemiluminescence (ECL) substrate (Beyotime, China) and imaged with an ECL western blot detection system (Millipore, Billerica, MA, USA) and normalized to GAPDH as a loading control.

### 2.18. Statistical Analysis

All data were analyzed using one‐way analysis of variance (ANOVA) and Student’s *t*‐test with SPSS 27.0 software (SPSS, Inc., Chicago, IL, USA). Results are expressed as mean ± standard deviation (SD), and statistical significance was determined at a threshold of *p*‐value <0.05.

## 3. Results

### 3.1. Enhanced Mast Cell Activation and Degranulation at DFU Sites

To elucidate the role of immune cells in the progression of DFU, we employed CIBERSORT algorithm to quantify the proportions of 22 immune cell subtypes in each sample, as shown in Figure 1A. In DFS specimens, the top five immune cell populations were resting mast cells, M2 macrophages, resting memory CD4^+^ T cells, follicular helper T cells (Tfh), and resting dendritic cells. In contrast, the top five immune cell populations in DFU specimens were resting memory CD4^+^ T cells, resting mast cells, M2 macrophages, M0 macrophages, and activated mast cells. Differential analysis (Figure 1B) revealed significantly higher proportions of activated mast cells (red frame, *p*  < 0.01) and neutrophils (*p* < 0.05) in DFU compared to DFS, while activated NK cells (*p* < 0.01) and CD8^+^ T cells (*p* < 0.05) were markedly reduced in DFU. These observations indicate that DFU lesions are characterized by increased neutrophil infiltration and mast cell activation, coupled with reduced NK cell activity and CD8^+^ T cell abundance, which may contribute to impaired immune regulation and delayed wound healing in diabetic ulcers.

Figure 1Mast cell activation in DFU tissues and the inhibitory effects of ZZO. (A) Bioinformatic deconvolution (CIBERSORT) of 22 immune cell types in each specimen from the GSE143341 dataset. Scale bars: 50 μm. (B) A box plot displays the proportion of 22 types of immune cells in DFS group and DFU group, with statistical significance tested using the Wilcoxon rank‐sum test. A *p*‐value less than 0.05 indicates a statistically significant difference between the groups. (C) The representative images of H&E staining and TB staining for normal skin tissues, DFU tissue samples, and DFU tissues treated with ZZO. Black arrows indicate mast cells and red arrows indicate cytoplasmic granules released during mast cell degranulation. Scale bars: 50 μm. (D) A bar graph quantifies the number of degranulated mast cells per view in each group (*n*, normal = 6, DFU = 46, and DFU + ZZO = 46). Statistical analysis was performed using one‐way ANOVA;  ^∗^ *p* < 0.05,  ^∗∗^ *p* < 0.01 was considered statistically significant.

**Figure:**
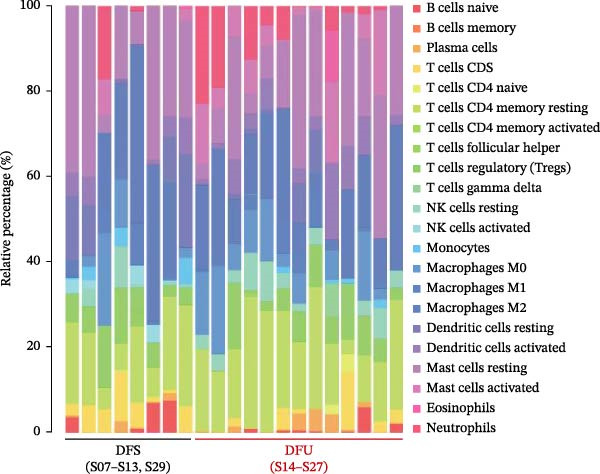
(A)

**Figure:**
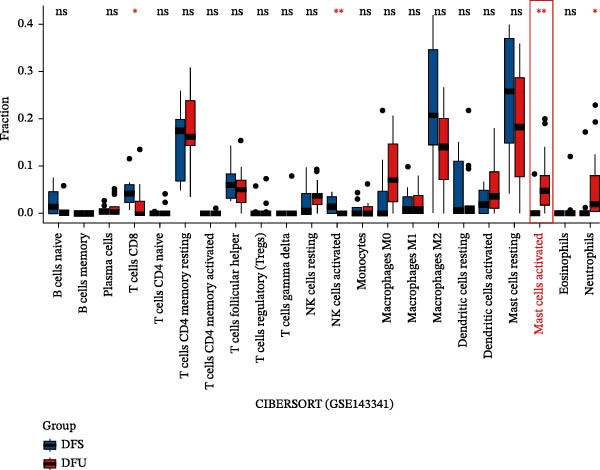
(B)

**Figure:**
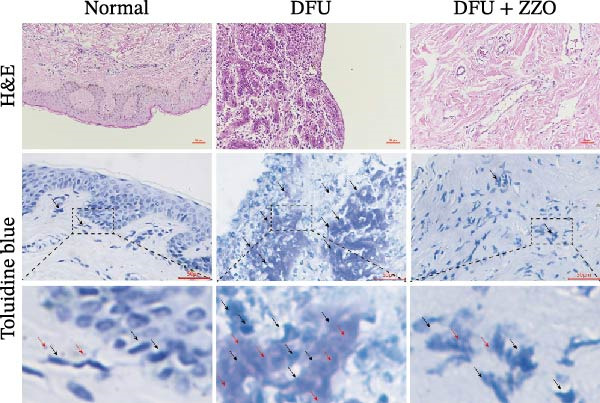
(C)

**Figure:**
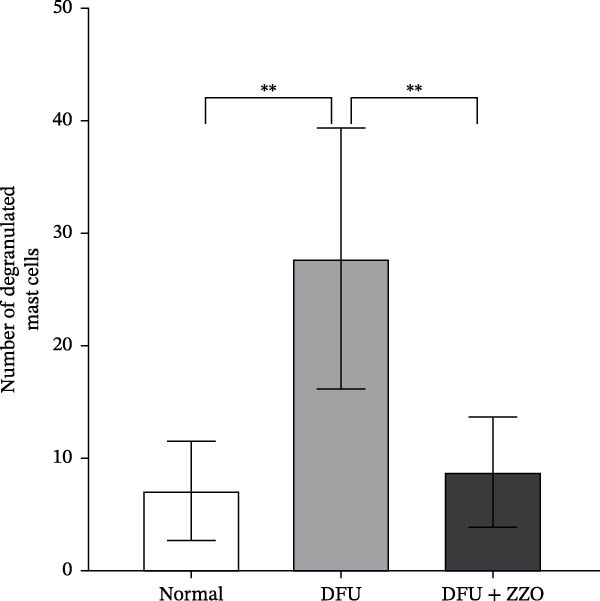
(D)

To evaluate mast cell activation in DFU tissues, all clinical specimens underwent H&E staining and TB staining (Figure 1C). H&E staining revealed significant infiltration of inflammatory cells in DFU lesions compared to normal skin, whereas treatment with ZZO significantly reduced the accumulation of these inflammatory cells. TB staining showed marked mast cell degranulation, characterized by extracellular metachromatic granules, in DFU tissues relative to normal controls. This degranulation was substantially diminished following ZZO therapy.

### 3.2. ZZO Promotes Wound Healing in Diabetic Mice

Results from the wound healing experiment (Figure 2A,B) indicated that compared to the control group, the DW group exhibited significantly reduced wound healing rates (WHRs) on day 7 (d_7_, *p*  < 0.01) and day 14 (d_14_, *p*  < 0.01). In contrast, the DW + ZZO group demonstrated significant improvements in WHR on both d_7_ (*p* < 0.01) and d_14_ (*p* < 0.01) compared to the DW group (Figure 2B).

Figure 2ZZO promotes wound healing in diabetic mice. (A) Representative wound images from each experimental group (*n* = 3 mice per group) at 0, 3, 7, and 14 days after treatment. (B) Quantitative analysis of wound healing rates (WHR) in each group at 3, 7, and 14 days post‐intervention. Data are presented as mean ± SD. Statistical analysis was performed using one‐way ANOVA followed by Tukey’s post hoc test to compare differences among groups.  ^∗∗^ *p* < 0.01 was considered statistically significant.

**Figure:**
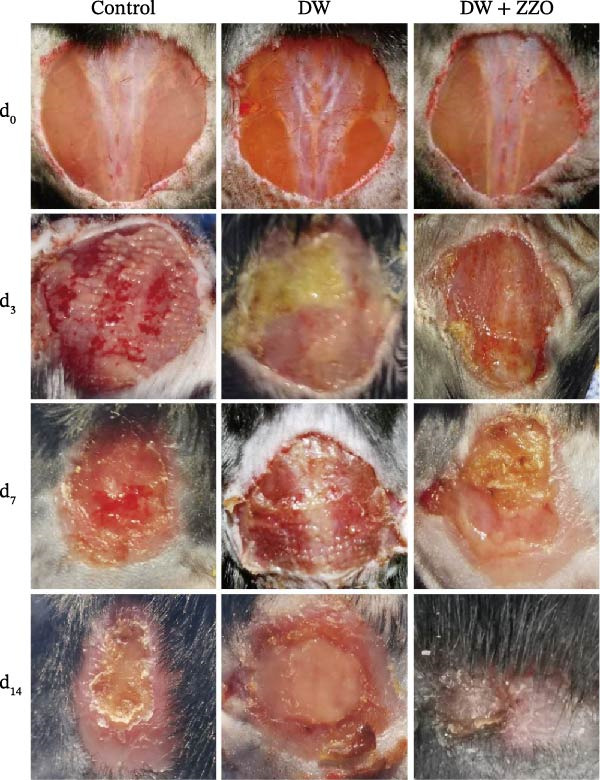
(A)

**Figure:**
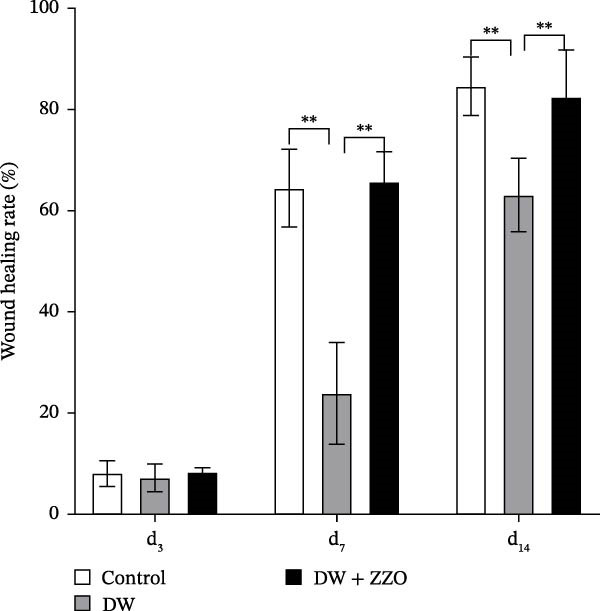
(B)

### 3.3. ZZO Inhibits Mast Cell Degranulation in Wounds of Diabetic Mice

Transcriptional profiling of wound tissues from diabetic model mice, utilizing CIBERSORT‐based analysis of 23 immune cell subsets, revealed distinct immune cell composition patterns. At day 3 post‐intervention (Figure 3A,B), the control group exhibited dominant infiltration of M0 macrophages (21.24%), M2 macrophages (19.65%), M1 macrophages (11.07%), monocytes (6.89%), and activated dendritic cells (6.34%). DWs (DW group) showed elevated proportions of M2 macrophages (29.66%), monocytes (18.11%), M1 macrophages (11.69%), neutrophils (7.33%), and naive B cells (6.46%). The DW + ZZO intervention group demonstrated a shifted profile dominated by M2 macrophages (15.59%), naive B cells (11.34%), naive CD4^+^ T cells (10.26%), M0 macrophages (8.92%), and M1 macrophages (8.69%).

Figure 3The impact of ZZO on the immune cell composition of diabetic mouse wounds. (A) Analysis of immune cell composition in mouse wound tissue at 3 days post‐intervention using transcriptome sequencing data, the CIBERSORT algorithm, and a mouse immune cell signature matrix. (B) Bar plot depicting differences in immune cell composition among control (D3C1), DW (D3DW1, D3DW2, and D3DW3), and DW + ZZO (D3DWZ1, D3DWZ2, and D3DWZ3) groups in murine wound tissue at 3 days post‐intervention. (C) Analysis of immune cell composition in mouse wound tissue at 7 days post‐intervention. (D) Bar plot depicting differences in immune cell composition among control (D7C1, D7C2, and D7C3), DW (D7DW1 and D7DW2), and DW + ZZO (D7DWZ1, D7DWZ2, and D7DWZ3) groups at 7 days post‐intervention.

**Figure:**
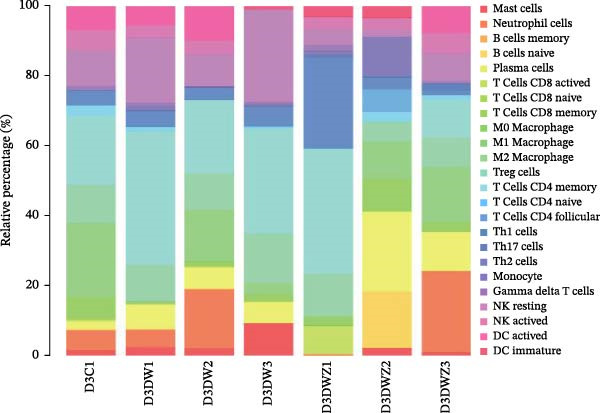
(A)

**Figure:**
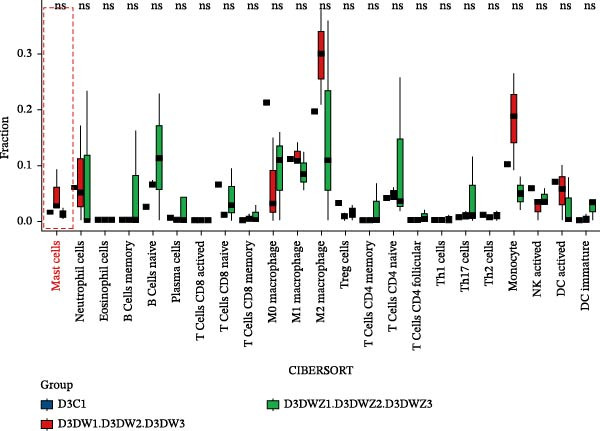
(B)

**Figure:**
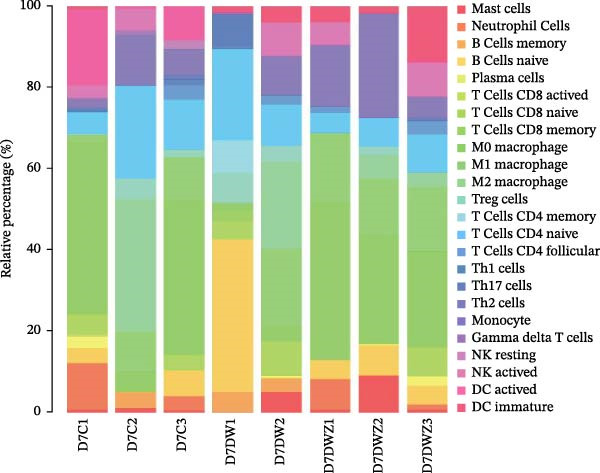
(C)

**Figure:**
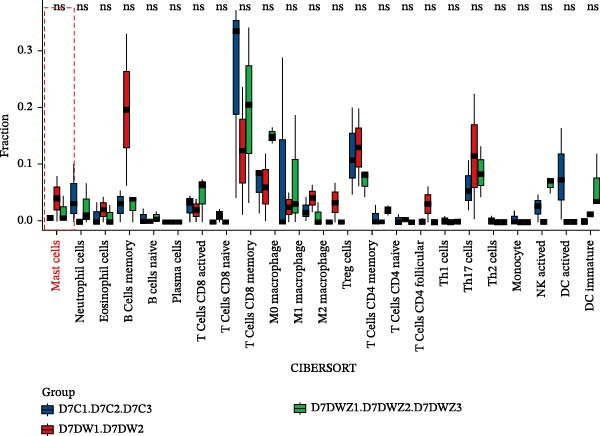
(D)

By day 7 (Figure 3C,D), control wounds maintained M0 macrophage predominance (28.26%) alongside naive CD4^+^ T cells (13.50%), M2 macrophages (10.89%), activated dendritic cells (9.16%), and M1 macrophages (7.35%). DWs displayed increased naive B cells (22.33%), naive CD4^+^ T cells (14.82%), M0 macrophages (14.16%), monocytes (13.12%), and M1 macrophages (6.96%). The DW + ZZO group exhibited rebalanced immune cell infiltration, marked by M0 macrophages (21.98%), M1 macrophages (17.20%), monocytes (9.88%), M2 macrophages (8.29%), and naive CD4^+^ T cells (8.21%).

Importantly, mast cell proportions exhibited a disease‐associated activation pattern: control groups had averages 1.46% (day 3) and 0.87% (day 7), whereas DWs showed elevated levels of 4.58% (day 3) and 4.69% (day 7). The ZZO intervention effectively reduced mast cell infiltration to 1.09% (day 3) and 2.25% (day 7), indicating a potential therapeutic modulation of mast cell activation. Due to limited sample sizes (*n* = 1–3 per group), formal statistical comparisons were precluded, though observed trends align with established DW pathophysiology.

The histopathological assessment of wound tissues revealed distinct inflammatory responses among the experimental groups. HE staining (Figure 4A) demonstrated significant infiltration of inflammatory cells in the diabetic model wounds compared to controls. Notably, ZZO‐treated wounds exhibited markedly reduced inflammatory cell accumulation relative to the model group, suggesting therapeutic attenuation of local inflammation. Complementary TB staining (Figure 4B) identified characteristic mast cell degranulation patterns. At post‐modeling days 3 and 7, model group wounds displayed abundant perimast cell granular dispersal, indicative of sustained activation. In contrast, control and ZZO‐treated groups maintained intact mast cell morphology (Figure 4C).

Figure 4ZZO inhibits mast cell degranulation in diabetic mouse wound tissue. (A) Representative H&E staining images of murine wound tissues showing epidermal and dermal architecture in each group. Scale bars: 50 μm. (B) Toluidine blue staining of mast cells in wound tissues from normal, diabetic, and ZZO‐treated diabetic mice. Degranulated mast cells are indicated by cytoplasmic granule dispersion. Black arrows indicate mast cells and red arrows indicate cytoplasmic granules released during mast cell degranulation. Scale bars: 50 μm. (C) Quantitative analysis of the number of degranulated mast cells per view in each group. Data are presented as mean ± SD from three biological samples per group (*n* = 3). Statistical analysis was performed using one‐way ANOVA followed by Tukey’s post hoc test;  ^∗∗^ *p* < 0.01 was considered statistically significant.

**Figure:**
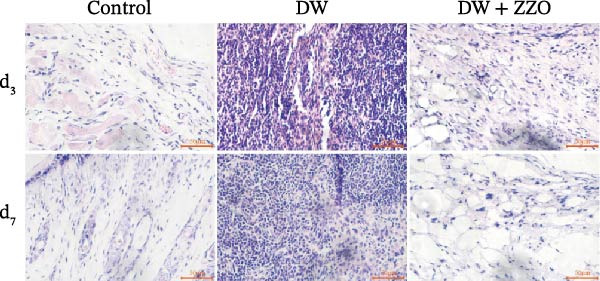
(A)

**Figure:**
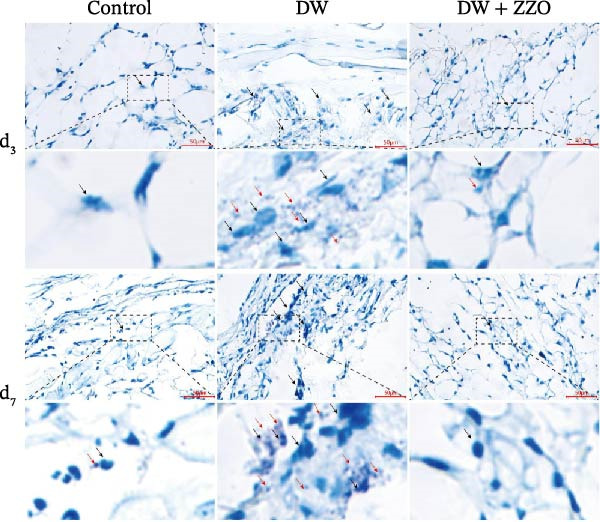
(B)

**Figure:**
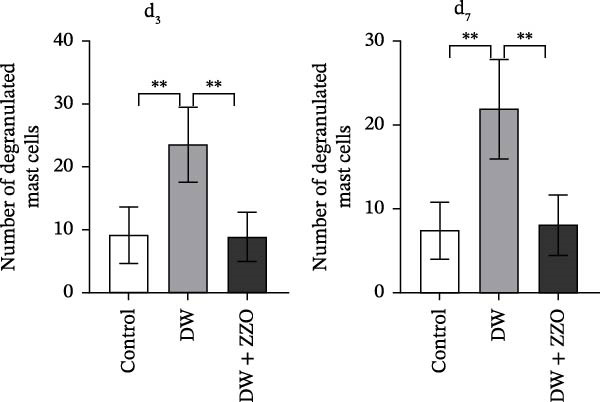
(C)

### 3.4. ZZO Inhibits the Expression of Inflammatory Factors MMP‐9 and TNF‐α in Wounds of Diabetic Mice

IHC analysis of DW tissues demonstrated significant alterations in mast cell‐derived inflammatory mediators. The expression of MMP9 (Figure 5A,B) and TNF‐α (Figure 5C,D) was markedly upregulated in the wounds of the model group compared to the control group at both day 3 and day 7. ZZO intervention substantially attenuated this inflammatory response, reducing TNF‐α and MMP9 expression to near‐control levels at day 3 and day 7. These findings mechanistically align with prior observations of mast cell stabilization, demonstrating ZZO‐mediated suppression of pro‐inflammatory factor secretion (TNF‐α) and ECM degradation (MMP9) in DWs.

Figure 5ZZO inhibits the expression of MMP‐9 and TNF‐α in wounds of diabetic mice. (A) Representative immunohistochemical (IHC) images showing MMP‐9 expression in wound tissues of diabetic mice at days 3 and 7 after treatment. Scale bars: 50 μm. (B) Quantitative analysis of relative MMP‐9 expression levels based on the mean optical density (MOD) values, normalized to the control group. (C) Representative IHC images showing TNF‐α expression in wound tissues of diabetic mice at days 3 and 7. Scale bars: 50 μm. (D) Quantitative analysis of relative TNF‐α expression levels based on MOD values, normalized to the control group. Data are expressed as mean ± SD from three mice per group (*n* = 3). Statistical significance was determined using one‐way ANOVA followed by Tukey’s post hoc test;  ^∗∗^ *p* < 0.01 was considered statistically significant.

**Figure:**
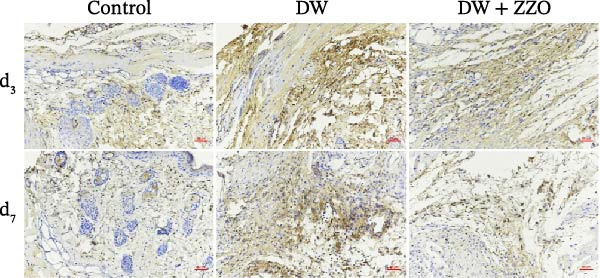
(A)

**Figure:**
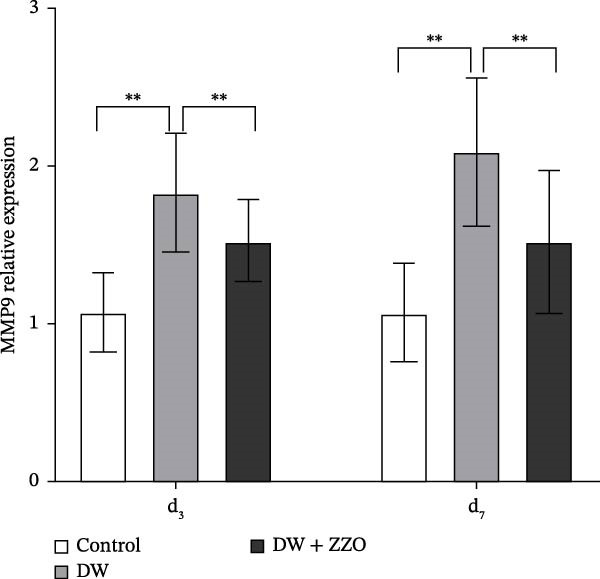
(B)

**Figure:**
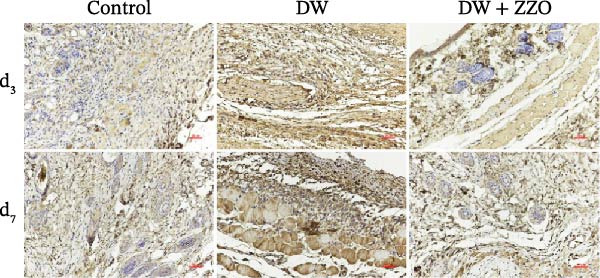
(C)

**Figure:**
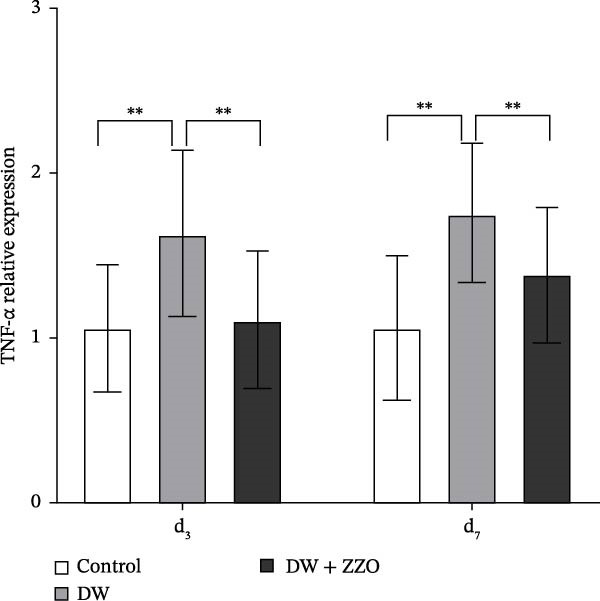
(D)

### 3.5. ZZO Suppresses IgE‐Triggered Activation and Degranulation in Both HMC‐1 Human and P815 Mouse Mast Cells

To evaluate the effect of ZZO directly on mast cells, the optimal working concentration was first determined. HMC‐1 and P815 cells were exposed to ZZO at concentrations of 50, 100, 200, 400, 800, 1600, and 3200 μg/mL, and cell viability inhibition rates were quantified using CCK‐8 assays. As shown in Figure 6A, the 10% inhibitory concentrations (IC_10_) of ZZO were determined to be 325.7 μg/mL for HMC‐1 cells and 295.2 μg/mL for P815 cells. Based on these results, 200 μg/mL—below the IC_10_ for both cell lines—was selected as the working concentration, ensuring minimal cytotoxicity while maintaining sufficient bioactivity. For all subsequent assays evaluating mast cell activation and degranulation, HMC‐1 and P815 cells were pretreated with 200 μg/mL ZZO for 1 h before IgE/DNP stimulation. Following pretreatment, mast cells were activated via IgE/DNP crosslinking in the presence or absence of 200 μg/mL ZZO.

Figure 6ZZO inhibits IgE‐mediated mast cell activation and degranulation. (A) Dose‐response curves of ZZO in HMC‐1 and P815 cells measured by CCK‐8 assays (*n* = 6). Dashed lines indicate IC_10_ values. (B) Representative toluidine blue staining of mast cell granules after IgE/DNP crosslinking with or without 200 μg/mL ZZO. Scale bars: 50 μmn. (C) Intracellular Ca^2+^ flux visualized by Fluo‐4 AM fluorescence. Scale bars: 50 μmn. (D) Flow cytometry histograms showing Ca^2+^ intensity in IgE‐activated cells with or without 200 μg/mL ZZO pretreatment. (E) Quantification of β‐hexosaminidase release ( ^∗∗^ *p* < 0.01). (F) Histamine concentrations in supernatants ( ^∗∗^ *p* < 0.01). (G) TNF‐α levels in supernatants ( ^∗∗^ *p* < 0.01).

**Figure:**
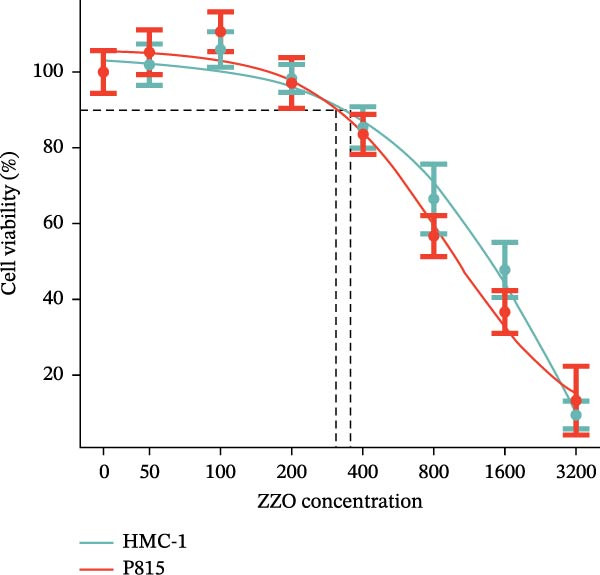
(A)

**Figure:**
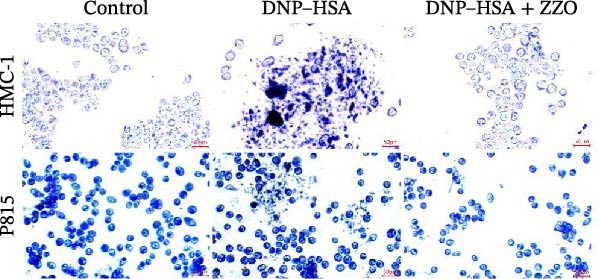
(B)

**Figure:**
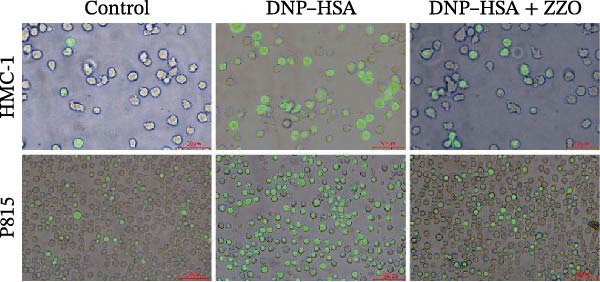
(C)

**Figure:**
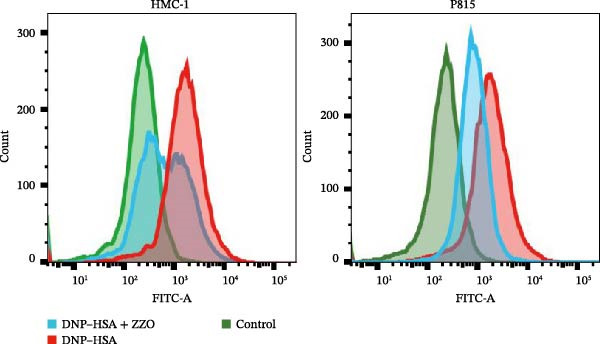
(D)

**Figure:**
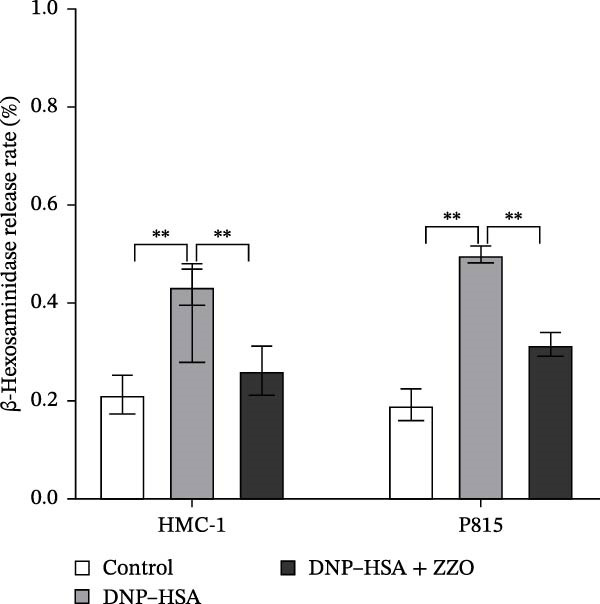
(E)

**Figure:**
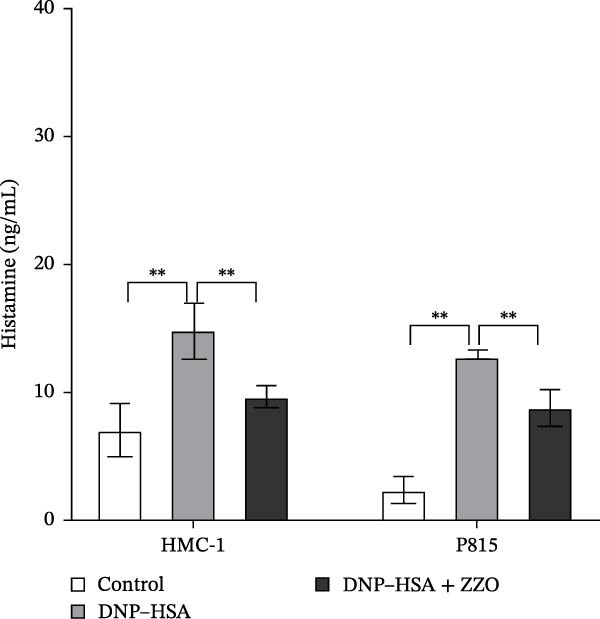
(F)

**Figure:**
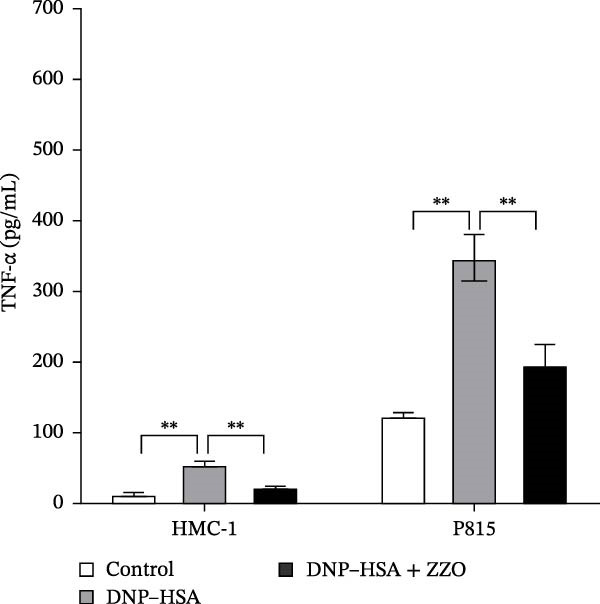
(G)

TB staining (Figure 6B) demonstrated that ZZO pretreatment markedly reduced IgE‐induced degranulation, as evidenced by decreased extracellular granule dispersion. In parallel, ZZO‐pretreated cells showed significantly reduced intracellular Ca^2+^ influx upon IgE challenge, as detected using Fluo‐4 AM fluorescence imaging (Figure 6C). Flow‐cytometric analysis further confirmed that ZZO attenuated surface activation markers associated with FcεRI‐mediated signaling (Figure 6D).

Consistently, functional assays demonstrated that ZZO pretreatment markedly inhibited multiple key indicators of mast cell degranulation. β‐hexosaminidase release was significantly reduced in the ZZO‐pretreated group compared with IgE/DNP‐activated cells (Figure 6E). In parallel, ELISA measurements confirmed that ZZO pretreatment suppressed the secretion of HIS (Figure 6F) and TNF‐α (Figure 6G) following IgE/DNP stimulation. Together, these findings demonstrate that pretreatment with 200 μg/mL ZZO effectively suppresses both the activation and degranulation pathways of mast cells triggered by IgE‐FcεRI crosslinking.

### 3.6. Modulatory Effects of ZZO on Mast Cell Activation Signaling and Associated Inflammatory Pathways

To elucidate the mechanism by which ZZO inhibits mast cell degranulation, IgE/DNP‐crosslinked HMC‐1 cells were treated with increasing concentrations of ZZO (50, 100, and 200 μg/mL). Western blot analysis revealed that ZZO dose‐dependently inhibited phosphorylation of critical signaling molecules: in FcεRI‐proximal activation pathways, it reduced p‐PI3K, p‐Akt, p‐PLCγ1, p‐SYK, and p‐LYN levels (Figure 7A); concomitantly, in inflammatory cascades, it attenuated phosphorylation of p‐IKKα/β, p‐NF‐κB, p‐p38, p‐JNK, and p‐ERK (Figure 7B). These concentration‐dependent impairments collectively demonstrate that ZZO disrupts both proximal activation signals and downstream effector pathways essential for mast cell degranulation.

Figure 7ZZO suppresses IgE‐mediated signaling pathways in mast cells. (A) Western blot analysis of FcεRI‐proximal activation markers in IgE/DNP‐crosslinked HMC‐1 cells co‐treated with ZZO (50, 100, or 200 μg/mL). Phosphorylated proteins (p‐PI3K, p‐Akt, p‐PLCγ1, p‐SYK, and p‐LYN) and corresponding total proteins were detected. GAPDH served as loading control. (B) Western blot analysis of inflammatory signaling mediators under identical treatment conditions. Phosphorylation status of IKKα/β, NFκB, p38, JNK, and ERK (with total protein controls) is shown.

**Figure:**
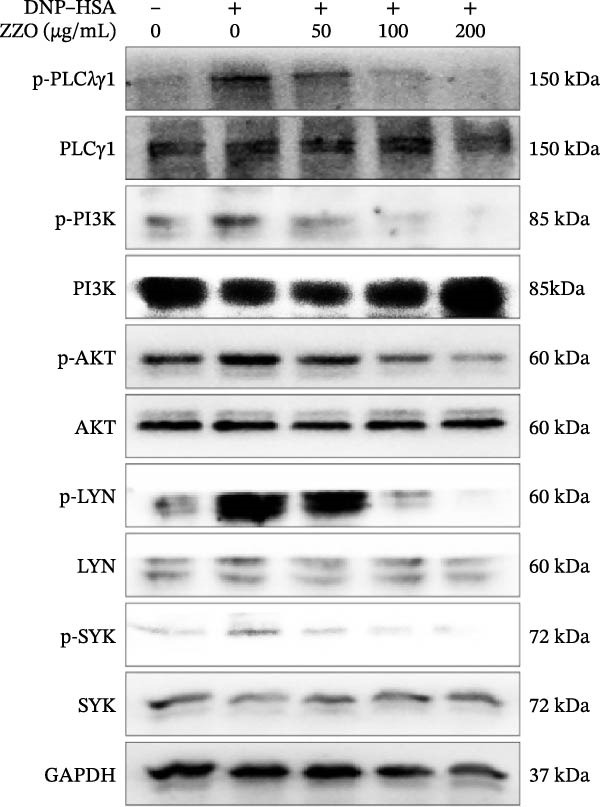
(A)

**Figure:**
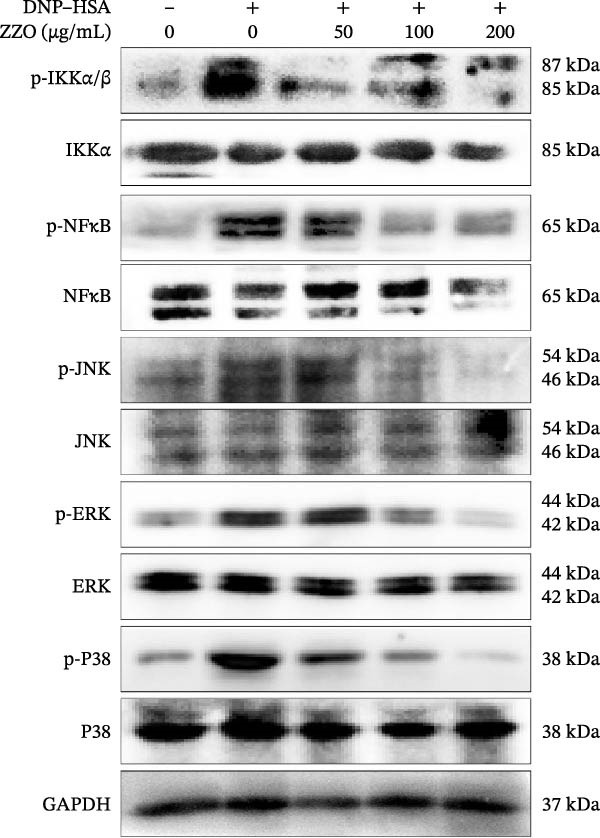
(B)

## 4. Discussion

DFU represents a particularly challenging complication associated with diabetes mellitus, characterized by impaired wound healing capabilities [23]. The underlying pathophysiological mechanisms primarily involve dysregulated activation of innate immune cells, with mast cells, neutrophils, and macrophages playing pivotal roles [24, 25]. This aberrant activation results in the continuous release of pro‐inflammatory mediators. Therefore, a self‐perpetuating inflammatory phase is established, while the proliferative phase essential for wound repair is concomitantly suppressed [26].

Mast cells, acting as sentinel cells within tissues [27], undergo pathological activation within DFU lesions [7]. Their persistent degranulation leads to the release of chemokines, such as CCL2 and CXCL8, which in turn recruit additional leukocytes to the site. Notably, mast cell‐derived TNF‐α and IFN‐γ are instrumental in driving the polarization of macrophages towards an M1 phenotype. These M1 macrophages subsequently secrete a array of inflammatory mediators, such as IL‐1β, IL‐6, and MMP‐9, which further amplify the inflammatory cascade and exacerbate tissue damage. Consequently, the pharmacological stabilization of mast cells, aimed at curbing their degranulation, presents itself as a promising therapeutic avenue. This strategy has the potential to disrupt the aforementioned vicious cycle and restore the physiological trajectories of wound healing in DFUs [7, 8, 13].

In the current study, we conducted an immunoinfiltration analysis using bioinformatics methods on the GSE134431 dataset sourced from the GEO database. Our findings indicate a statistically significant increase in both the number and activation of mast cells in DFU tissues, a finding that is in accordance with several preceding studies [7, 13, 15]. For example, Dong et al. [7] demonstrated evident mast cell degranulation in DFU wounds. To further substantiate these findings and explore therapeutic efficacy, we utilized DFU specimens from a previous ZZO clinical study, comparing them with normal skin specimens. Previous clinical evidence suggests that ZZO can accelerate DFU healing and reduce the infiltration of inflammatory cells as well as the levels of inflammatory factors in ulcer tissues [18, 28, 29]. In our validation endeavors, TB staining unveiled extensive mast cell degranulation in the ulcer tissues of DFU patients, a phenomenon that was significantly attenuated by ZZO treatment. This study not only confirms the aberrant changes in mast cell profiles within DFU tissues but also unveils the potential of ZZO in modulating mast cell behavior. These findings are significant as they provide a foundation for the development of novel DFU treatment strategies that specifically target mast cells.

Emerging evidence suggests that mast cell stabilizers, such as cromolyn sodium (DSCG) [8] and MCS‐01 [30], can enhance wound healing in diabetic mouse models. Within our study, utilizing the STZ‐induced diabetic mouse wound model, we observed that ZZO administration facilitated wound healing in DWs. Although the sample size was relatively small and the differences between groups did not reach statistical significance, immune cell composition analysis indicated that ZZO might suppress mast cell activation within the diabetic ulcer model. Consistent with clinical specimens, mast cell degranulation was evident in the tissues of the diabetic ulcer model; however, ZZO treatment resulted in a reduction of this degranulation. Furthermore, the levels of MMP‐9 and TNF‐α were elevated in diabetic ulcer tissues but were lowered in the ZZO‐treated group. Considering that these inflammatory factors serve as primary mediators released subsequent to mast cell activation, it can be inferred that ZZO may facilitate its positive effects on DW healing by inhibiting mast cell activation and consequently reducing the secretion of inflammatory factors. These findings hold considerable importance for future research exploring the potential of ZZO in DW treatment and for the development of innovative therapies targetting mast cells.

Within the local microenvironment of DFUs, numerous factors contribute to the persistent activation of mast cells, including IgE and allergens, hyperglycemia, tryptase, inflammatory cytokines—such as IL‐1β and IL‐6—chemokines, and complement components [15, 25]. The IgE/DNP cross‐linking model is one of the commonly utilized experimental models for studying mast cell activation [31–33]. In our study, we observed that IgE/DNP cross‐linking activated HMC‐1 and P815 cells, leading to significant mast cell degranulation. However, ZZO treatment proved effective in inhibiting this degranulation process. Moreover, the combination of Ca^2+^ ion staining, β‐hexosaminidase release assays, and the quantification of HIS and TNF‐α levels collectively substantiates that ZZO effectively inhibits mast cell activation. This indicates its potential in modulating mast cell function and suggests its possible application in the treatment of inflammatory diseases.

Upon IgE binding to the FcεRI receptor on mast cells, allergen entry prompts the cross‐linking of IgE‐bound FcεRI, thus activating the receptor. The FcεRI receptor is composed of an α subunit that binds IgE, along with β and γ subunits containing immunoreceptor tyrosine‐based activation motifs (ITAMs). Subsequently, Lyn kinase phosphorylates these ITAMs, leading to the activation of Syk kinase. Syk then phosphorylates PLCγ1, which hydrolyzes PIP2 into IP3 and DAG. IP3 facilitates an increase in cytosolic Ca^2+^ levels, causing Ca^2+^ influx. Concurrently, PI3K converts PIP2 into PIP3, activating Akt. Additionally, FcεRI activation initiates the IKK/NFκB and MAPK (p38, JNK, and ERK) pathways. NFκB activates the transcription of inflammatory genes, while MAPKs manage inflammation and stress responses. Collectively, these pathways orchestrate mast cell degranulation, leading to the release of HIS and cytokines [34–36]. In this study, we found that ZZO inhibits the IgE/DNP cross‐linking mediated activation of the PI3K/AKT, SYK/LYN/PLCγ1, and IKK/NFκB signaling pathways, as well as the P38/JNK/ERK pathways. Based on pathway hierarchy, Lyn/Syk phosphorylation represents the proximal (upstream) event following FcεRI activation, while PI3K/Akt and NF‐κB are downstream effectors that integrate multiple receptor‐driven signals to regulate gene transcription and degranulation. Therefore, our data suggest that ZZO primarily interferes with the early Lyn/Syk–PLCγ1 axis, which in turn leads to secondary suppression of downstream PI3K/Akt and NF‐κB signaling. This hierarchical inhibition provides a mechanistic explanation for how ZZO attenuates mast cell activation and degranulation by simultaneously dampening both upstream receptor phosphorylation and downstream inflammatory amplification. This indicates that ZZO suppresses mast cell activation and degranulation by inhibiting these relevant signaling cascades.

ZZO, a topical formulation derived from TCM, integrates six herbal components: cinnabaris, *Arnebia euchroma*, *Astragalus membranaceus*, *Dracaena cochinchinensis* resin, *Equus asinus gelatin*, and borneol. The clinical findings of this study, together with previous clinical trials, demonstrate that topical ZZO provides a significant therapeutic benefit in patients with DFU. ZZO not only accelerates wound closure but also reduces ulcer area and depth, alleviates pain, and improves local microcirculatory perfusion. These results are consistent with earlier evidence showing that ZZO is effective in various types of chronic lower‐limb ulcers, suggesting its broad clinical applicability in chronic, inflammation‐driven wounds [37–39]. In our previous study, UPLC‐HRMS profiling was performed to chemically characterize and standardize the ZZO extract, ensuring batch‐to‐batch reproducibility and analytical consistency. A total of 21 major bioactive constituents were identified as the most abundant compounds within the formulation [18]. Among these, four key constituents—eucalyptol, pinocembrin, isovalerylshikonin, and daidzein—have been previously reported to exert mast cell–stabilizing or anti‐inflammatory effects [18, 40–43]. Specifically, eucalyptol inhibits SYK and LYN [40], pinocembrin and isovalerylshikonin blocks NFκB signal [41], and daidzein inhibits PI3K/AKT signal [43] and TNF‐α/IL‐6 secretion [44]. This multicomponent system acts in synergy to attenuate FcεRI‐proximal signaling (LYN/SYK/PLCγ), downregulate inflammatory cascades (IKK/NF‐κB and MAPKs), and inhibit degranulation and mediator release. Thus, this provides a mechanistic foundation for ZZO’s clinical efficacy in DFU treatment through mast cell stabilization and inflammation resolution.

In conclusion, our integrated study establishes that ZZO addresses the core pathophysiology of DFU by disrupting mast cell‐driven inflammatory cascades. Through multimodal inhibition of FcεRI‐proximal signaling (LYN/SYK/PLCγ/PI3K‐Akt) and downstream effectors (IKK/NFκB and MAPKs), ZZO significantly suppresses degranulation. Collectively, these findings provide a robust rationale for developing mast cell‐targeted DFU therapies and highlight ZZO as a promising treatment candidate.

## Funding

This study was funded by the National Natural Science Foundation of China (Grant 82274528), the Scientific Research Project of Shanghai Municipal Health Commission (Grant 202240228), and the Science and Technology Development Fund Project of Shanghai University of Traditional Chinese Medicine (Grant 24KFL070).

## Conflicts of Interest

The authors declare no conflicts of interest.

## Data Availability

The data that support the findings of this study are available from the corresponding author upon reasonable request.
